# Supplementary material for: Co-Expression Networks in Sunflower: Harnessing the Power of Multi-Study Transcriptomic Public Data to Identify and Categorize Candidate Genes for Fungal Resistance
Source: Plants (Basel). 2023 Jul 25;12(15):2767. doi: 10.3390/plants12152767 (PMC10421300; doi:10.3390/plants12152767)
Supplement: Supplementary file 1 [file plants-12-02767-s001.zip › Supplementary Data.pdf]

Co-expression networks in sunflower: harnessing the power of multi-study transcriptomic public data to identify and categorize candidate genes for fungal resistance

Andrés Ribone, Mónica Fass, Sergio Gonzalez, Veronica Lia, Norma Paniego and Máximo Rivarola

Supplementary Figures

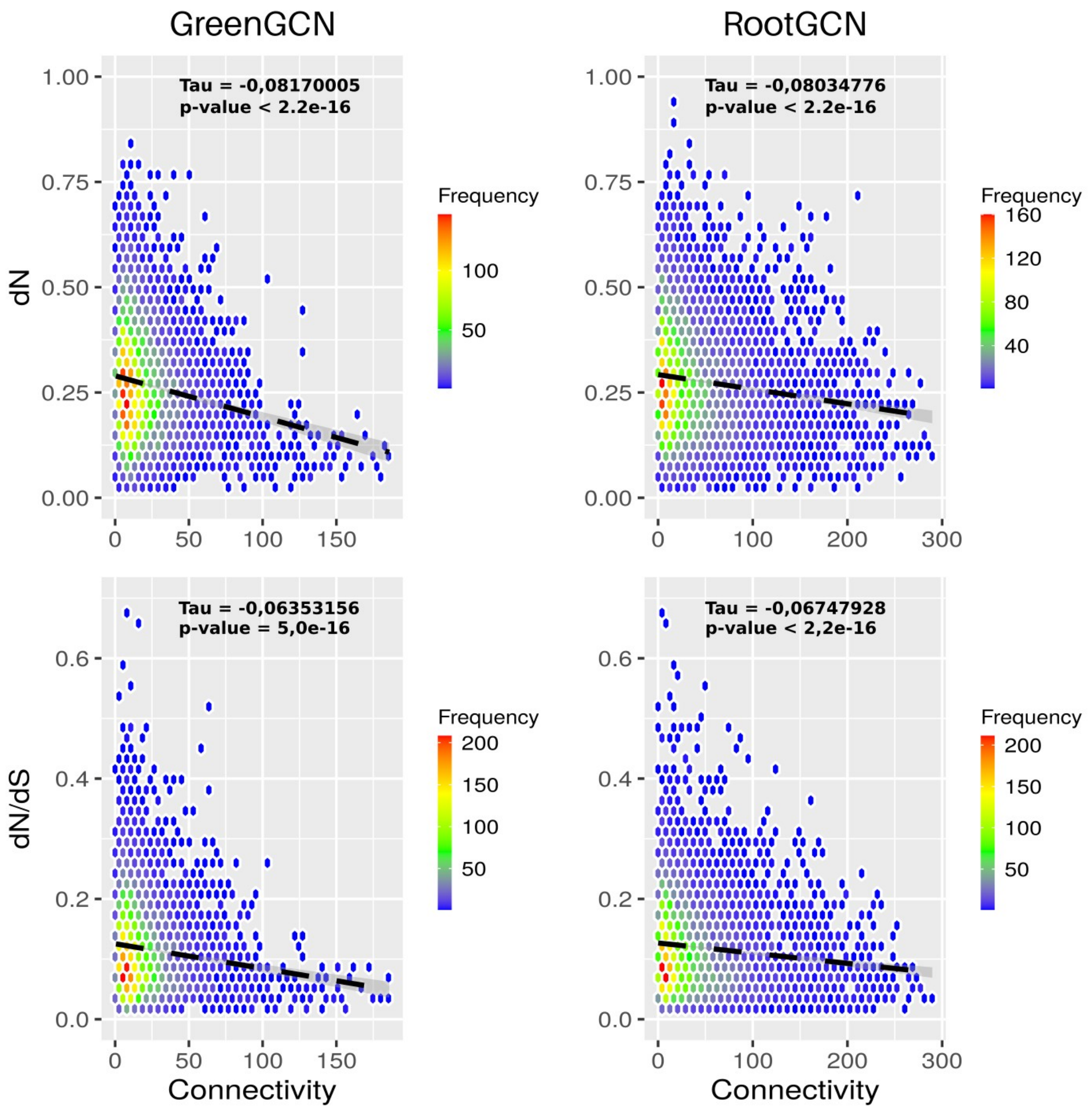

Figure S1. Gene connectivities versus molecular evolution rates (MER), with Kendall Rank tests. MERs were measured only in genes with putative orthologs in *Arabidopsis thaliana* via Best Reciprocal BlastP Hits: 7251 in GreenGCN and 8032 in RootGCN.

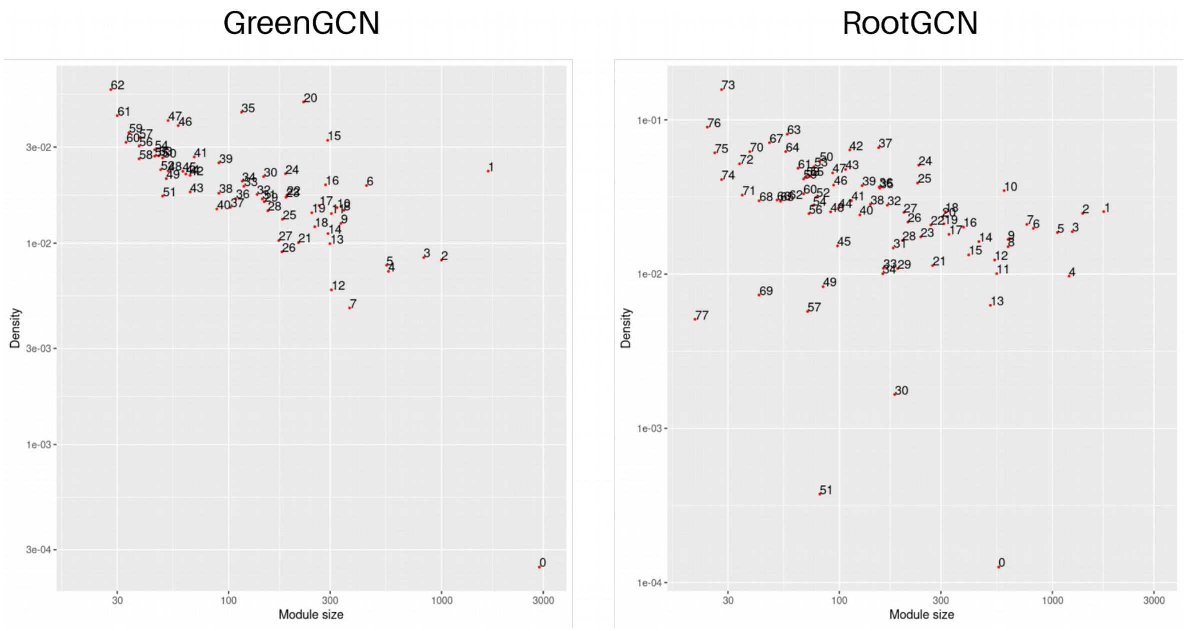

Figure S2. Module densities versus size in each GCN.

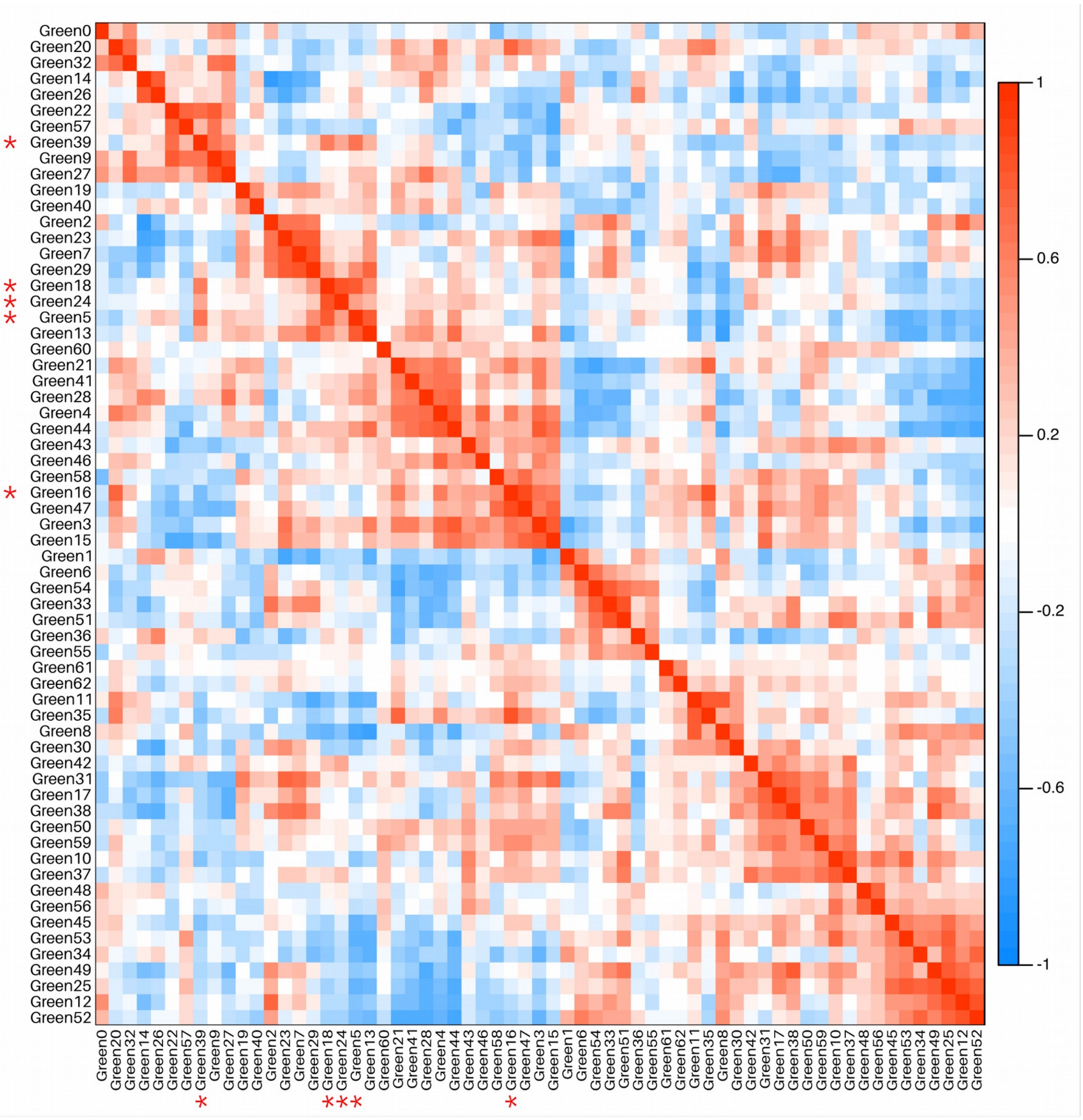

Figure S3. Heatmap of correlations between modules eigengenes from the GreenGCN. Asterisks note our modules of interest.

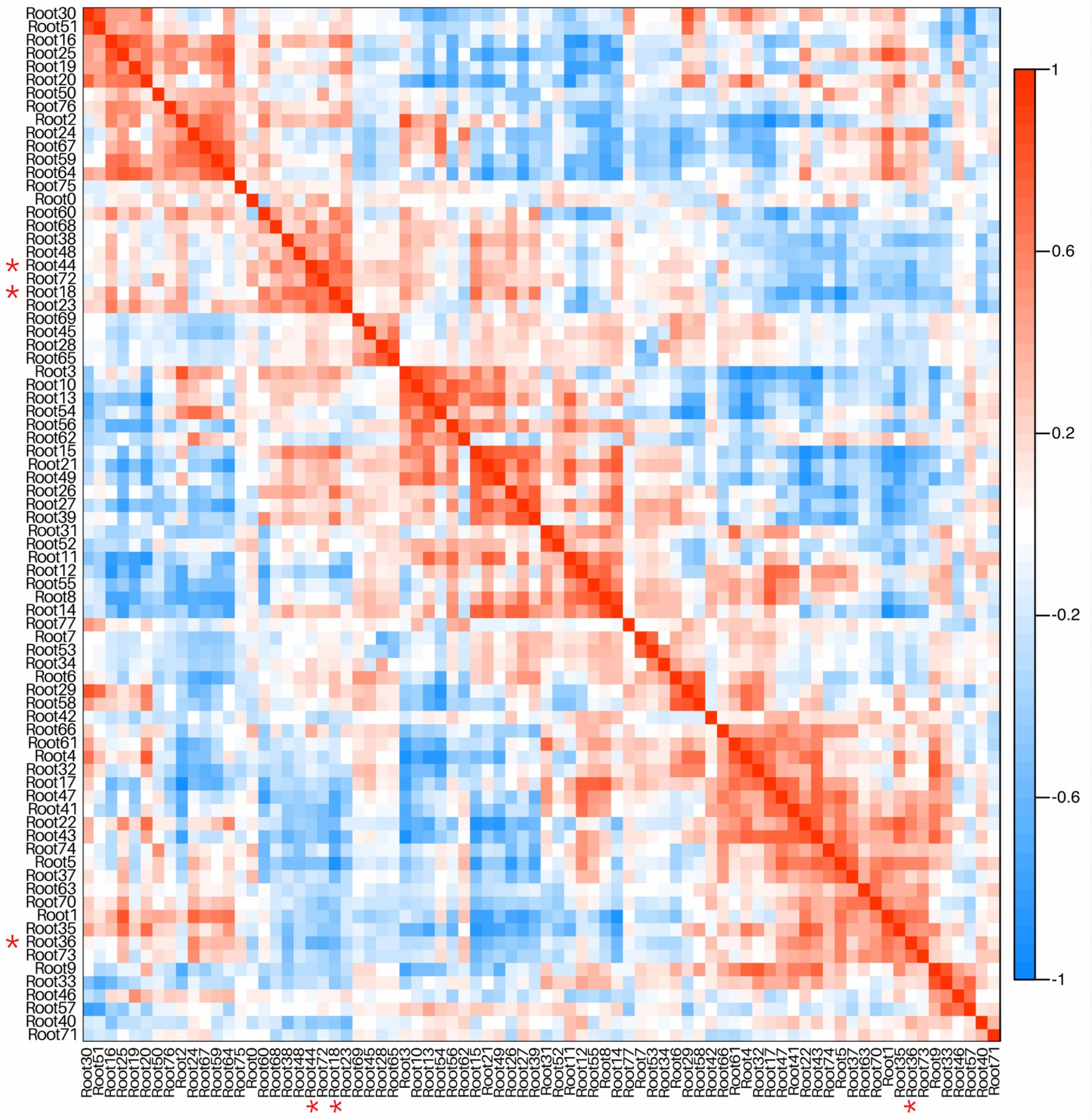

Figure S4. Heatmap of correlations between modules eigengenes from the RootGCN. Asterisks note our modules of interest.

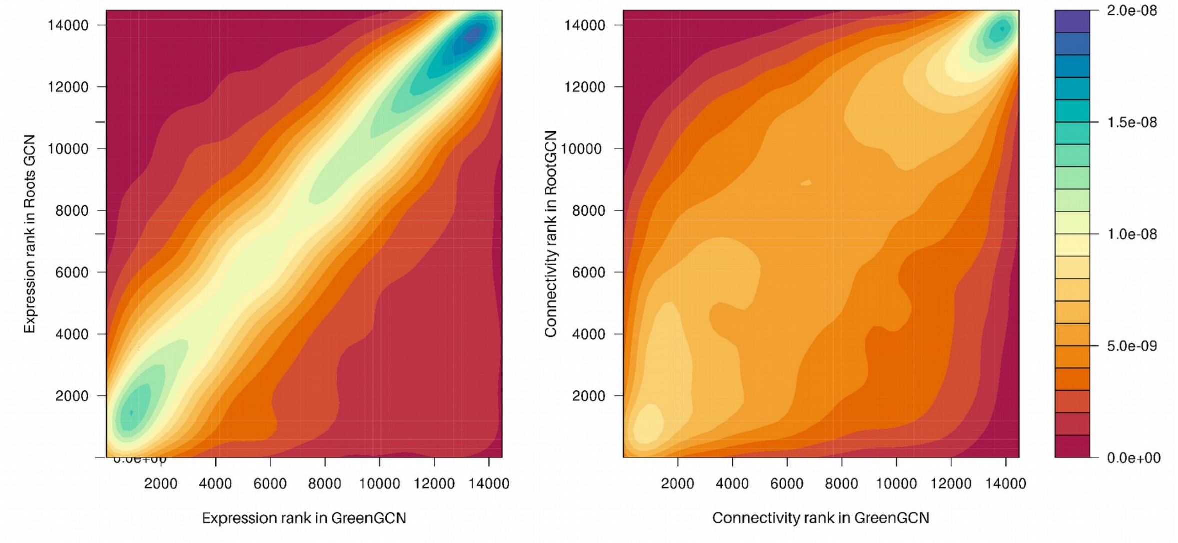

Figure S5. Comparison of expression rank (left) and connectivity rank of genes shared between both GCNs. Pearson correlations are 0.68 (p-value < 1E-200) and 0.5 (p-value < 1E-200) respectively.

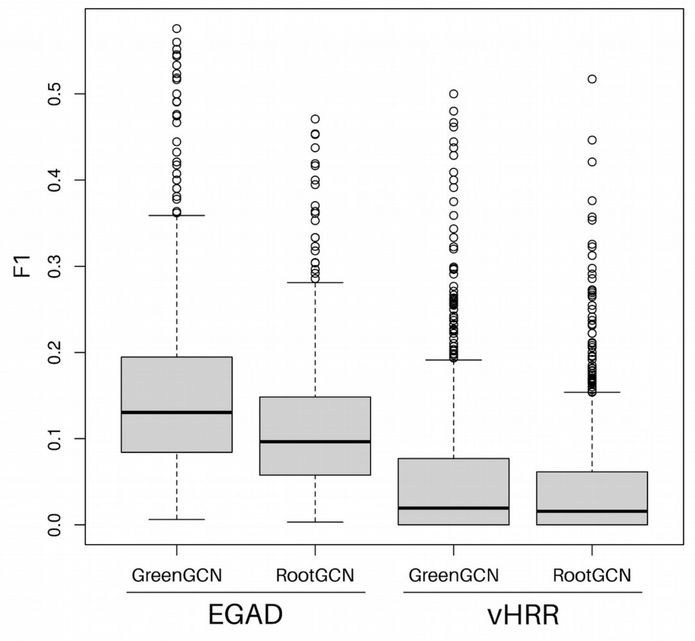

Figure S6. Comparison of prediction performance of EGAD and vHRR.

Supplementary Tables

Table S1. Synonymy between WRKY genes from different studies. Cross symbols (†) indicate genes present in modules of interest in the present work. Asterisks (*) indicate genes differentially expresed against pathogens in each corresponding study. Ellipsis (…) indicate genes that couldn't be identified in each study.

Gene name<br>Present work (HanXRQ1.2)	Giacomelli et al. (2010)	Liu et al. (2020)	Li et al. (2020)	Module
HanXRQChr16g0499381 †	HaWRKY2 *	HaWRKY105	HaWRKY81	Green24 / Root18
HanXRQChr08g0216831 †	HaWRKY3 *	HaWRKY045	HaWRKY43	Green24 / Root44
HanXRQChr03g0088861 †	HaWRKY4 *	HaWRKY014	…	Green24 / Root23
HanXRQChr16g0509771 †	HaWRKY7 *	HaWRKY109	…	Green24 / Root48
HanXRQChr16g0505941 †	HaWRKY10 *	HaWRKY106	HaWRKY79	Green39 / Root18
HanXRQChr03g0084521 †	HaWRKY76 *	HaWRKY011	…	Green24 / Root2
HanXRQChr14g0460611 †	HAWRKY44	HaWRKY090 *	…	Green18 / Root44
HanXRQChr15g0480431 †	…	HaWRKY101 *	HaWRKY75	Green5 / Root18
HanXRQChr11g0348481 †	HAWRKY63	HaWRKY081 *	…	Root18
HanXRQChr17g0533851	HaWRKY5 *	HaWRKY114 *	…	Root60
HanXRQChr09g0244461	HaWRKY75 *	HaWRKY048	HaWRKY33	Root2
HanXRQChr05g0128791	HaWRKY8 *	HaWRKY023	HaWRKY62	-
HanXRQChr04g0127081	…	HaWRKY022 *	HaWRKY15 *	-
HanXRQChr08g0232861	HAWRKY95	HaWRKY047 *	HaWRKY30	Green0 / Root1
HanXRQChr10g0306421	HAWRKY27	HaWRKY073 *	HaWRKY48	Green0 / Root9
HanXRQChr03g0085191	…	HaWRKY012 *	HaWRKY9	-
HanXRQChr05g0158011	…	HaWRKY027 *	…	Root1
HanXRQChr06g0166011	HAWRKY85	HaWRKY029 *	HaWRKY18	-
HanXRQChr06g0166061	HAWRKY82	HaWRKY030 *	…	-
HanXRQChr07g0191161	...	HaWRKY037 *	…	-
HanXRQChr07g0191171	HAWRKY62	HaWRKY038 *	HaWRKY22	-
HanXRQChr13g0408021	HAWRKY66	HaWRKY087 *	HaWRKY58	Root4
HanXRQChr14g0427721	...	HaWRKY088 *	HaWRKY70	Root7
HanXRQChr10g0290491	HAWRKY18	HaWRKY069	HaWRKY45 *	-
HanXRQChr10g0304971	HAWRKY86	HaWRKY072	HaWRKY44 *	-
HanXRQChr15g0464761	HAWRKY45	HaWRKY096	HaWRKY68 *	-
HanXRQChr15g0480811	...	HaWRKY102	HaWRKY76 *	Root27
HanXRQChr16g0514201	...	HaWRKY110	HaWRKY85 *	-
HanXRQChr15g0468081	HAWRKY32	HaWRKY099	HaWRKY71 *	Green13 / Root8
HanXRQChr03g0082581	…	HaWRKY010	HaWRKY7 *	-
